# Rituximab identified as an independent risk factor for severe PJP: A case-control study

**DOI:** 10.1371/journal.pone.0239042

**Published:** 2020-09-11

**Authors:** Anat Zalmanovich, Ronen Ben-Ami, Galia Rahav, Danny Alon, Allon Moses, Karen Olshtain-Pops, Miriam Weinberger, Pnina Shitrit, Michal Katzir, Bat-Sheva Gottesman, Michal Chowers

**Affiliations:** 1 Department of Internal Medicine A, Meir Medical Center, Kfar Saba, Israel; 2 Sackler School of Medicine, Tel Aviv University, Tel Aviv, Israel; 3 Infectious Disease Unit, Tel Aviv Medical Center, Tel Aviv, Israel; 4 Infectious Disease Unit, Sheba Medical Center, Ramat Gan, Israel; 5 Infectious Disease Unit, Hadassah Medical Center, Jerusalem, Israel; 6 Hebrew University of Jerusalem, Jerusalem, Israel; 7 Infectious Disease Unit, Assaf Harofeh Medical Center, Be’er Yaakov, Israel; 8 Infectious Diseases Unit, Meir Medical Center, Kfar Saba, Israel; University of Texas McGowan Medical School at Houston, UNITED STATES

## Abstract

**Objective:**

*Pneumocystis jirovecii* pneumonia (PJP) was reported among immunosuppressed patients with deficits in cell-mediated immunity and in patients treated with immunomodulatory drugs. The aim of this study was to identify risk-factors for PJP in noninfected HIV patients.

**Methods:**

This retrospective, test negative, case-control study was conducted in six hospitals in Israel, 2006–2016. Cases were hospitalized HIV-negative patients with pneumonia diagnosed as PJP by bronchoalveolar lavage. Controls were similar patients negative for PJP.

**Results:**

Seventy-six cases and 159 controls were identified. Median age was 63.7 years, 65% males, 34% had hematological malignancies, 11% inflammatory diseases, 47% used steroids and 9% received antilymphocyte monoclonal antibodies. PJP was independently associated with antilymphocyte monoclonal antibodies (OR 11.47, CI 1.50–87.74), high-dose steroid treatment (OR 4.39, CI 1.52–12.63), lymphopenia (OR 8.13, CI 2.48–26.60), low albumin (OR 0.15, CI 0.40–0.54) and low BMI (OR 0.80, CI 0.68–0.93).

**Conclusion:**

In conclusion, rituximab, which is prescribed for a wide variety of malignant and inflammatory disorders, was found to be significant risk-factor for PJP. Increased awareness of possible PJP infection in this patient population is warranted.

## Introduction

*Pneumocystis jirovecii* pneumonia (PJP) is an opportunistic fungal infection that mostly afflicts immunosuppressed patients with deficits in cell-mediated immunity [1]. PJP was first described in malnourished infants in Europe following World War II [2]. In the 1960s and 1970s, it was diagnosed primarily among patients with hematologic malignancies [3]. From the 1980s until the present, PJP is most often found in patients with advanced AIDS. Other known risk-factors include corticosteroid treatment for various indications [4], solid tumors [1, 5], inflammatory diseases [6], and immunosuppressive drug use following bone marrow and solid organ transplantation [7]. The introduction of active antiretroviral therapy and the use of cotrimoxazole prophylaxis resulted in a dramatic drop in the incidence of PJP among patients with HIV infection. Several studies have reported increased rates of PJP among patients treated with new immunomodulatory drugs (monoclonal antibodies, anti-TNF therapy, tyrosine kinase inhibitors and proteosome inhibitors) [8–12]. Patients in most of these studies were treated with combinations of immunosuppressive drugs; thus, it was impossible to separate the role of each one.

The prognosis of PJP among patients without HIV is typically worse than in those with HIV, with high rates of in-hospital mortality (50% to 86%, according to some reports [4, 13]), respiratory failure and ICU admission [14]. Due to a tendency for rapid and severe progression, early diagnosis and treatment of PJP is vital. Furthermore, since the diagnosis of PJP is usually established by bronchoalveolar lavage (BAL), a high level of suspicion is needed for early detection and initiation of appropriate treatment.

The aim of this study was to assess risk-factors for PJP among non-HIV patients.

## Methods

Study design: This retrospective, test-negative, case-control study was performed at 6 tertiary-level medical centers in Israel. All participating hospitals had a dedicated haemato-oncology department, 5 had stem cell transplantation units, and 5 had solid organ transplantation services. The 6 general hospitals had a total of 5,516 beds, among 15,871 in the entire country (35%). The study was conducted from January 2006 through December 2016.

Case definition: A case was defined as a hospitalized patient with clinical and radiological evidence of pneumonia and a positive direct microscopic examination for PJP (May-Grunwald-Giemsa stain, Gomori-Grocott staining or immunofluorescence) from BAL fluid. All PJP diagnosed by pathology were available, and electronic medical records were reviewed for evidence of pneumonia and HIV status. All cases diagnosed during the study period were followed until discharge.

Controls were defined as hospitalized patients with clinical and radiological evidence of pneumonia, for which PJP was excluded by BAL. Controls were matched 2:1 by age and sex to PJP cases. Exclusion criteria were younger than 18 years-of-age and HIV-positive status. HIV negativity was determined based on documentation in the medical records. To minimize false positive results representing PJP colonization, we did not include patients with PJP detected by PCR alone [15, 16].

Data extracted from the electronic medical records of cases and controls included demographics, past medical history, presence of an immunosuppressive condition considered a risk-factor for PJP (including hematological malignancies, solid tumors, solid organ transplant, bone marrow transplant and inflammatory disorders), use of immunosuppressant drugs (including corticosteroids with a dosage equivalent to ≥20 mg prednisone for ≥2 weeks, cytotoxic chemotherapy and immunomodulatory drugs) and use of trimethoprim/sulfamethoxazole as prophylactic agents. Laboratory results and clinical outcomes were collected, as well. Due to the large variety of risk-factors in the medical history and the large number of immunosuppressive drugs in use, we grouped drugs and diseases by mechanisms. The complete list is presented in S1 and S2 Tables.

Meir MC review board approved the study approval number 268–16. Tel-Aviv MC review board approved the study, Sheba MC review board approved the study, Hadassah MC review board approved the study, Assaf Harofeh MC review board approved the study, Rabin MC review board approved the study.

No consent was required, data were analyzed anonymously.

### Statistical analyses

Nominal parameters are presented as numbers and percentages. For nominal variables, the study and control groups were compared using Chi-square or Fisher’s exact test, as appropriate. For continuous variables, differences between the groups were determined using Student’s t-test or the Mann-Whitney non-parametric test, according to the distribution of the values. Multivariate logistic regression analysis was conducted including variables significantly associated with PJP on univariate analysis (p<0.1), selecting risk variables among correlated data. All data were analyzed using SPSS-25 software.

## Results

Seventy-six patients met the case definition for non-HIV related PJP and were compared to 159 matched control patients. The average age of both groups was 63 years. Most patients were men, 64% and 67% in the case and control groups, respectively. Demographic, clinical, laboratory and hospitalization outcome data for both groups are presented in Table 1.

**Table 1:** Demographics, medical history, drug, clinical, laboratory, imaging and hospitalization outcomes for case and control groups.

Variable		PJP Cases (N = 76)	Non-PJP Controls (N = 159)	P-value
Male sex, n (%)		49 (64.5)	109 (67.3)	0.661
Median (IQR) age (years)		66 (57–73)	64 (58–72)	0.713
Body mass index, kg/m^2^, median (IQR)		21 (19–23) (n = 47)	25 (22–27) (n = 114)	<0.001
Medical history, n (%)	Hematologic malignancies	34 (44.7)	46 (28.9)	0.017
	Inflammatory disease	14 (18.4)	11 (6.9)	0.007
	Solid tumor	14 (18.4)	18 (11.3)	0.138
	Organ transplant	7 (9.2)	33 (20.8)	0.028
Drugs, n (%)	Corticosteroids^a^	47 (61.8)	36 (22.6)	<0.001
	Chemotherapy	28 (36.8	31 (19.5)	0.006
	Rituximab	12 (15.8)	8 (5.0)	0.008
	Antimetabolites	8 (10.5)	6 (3.8)	0.072
	Immunomodulatory	2 (2.6)	5 (3.1)	0.594
	Immunosuppressive	10 (13.2)	33 (20.8)	0.207
	Kinase inhibitors	2 (2.6)	6 (3.8)	0.727
Preventive therapy for PJP, n (%)	Sulfamethoxazole and trimethoprim	5 (6.6)	26 (16.4)	0.038
Symptoms, n (%)	Cough	38 (50)	108 (67.9)	0.008
	Fever	56 (72.4)	115 (73.0)	0.925
	Dyspnea	58 (76.3)	110 (67.7)	0.257
Laboratory results	White blood cells, 10^6^/L	10.8 ± 20.7	15.8 ± 24.3	0.02
	Hemoglobin, g/L	10.7 ± 1.74	11 ± 1.96	0.187
	Lymphopenia, <1000/mcl, n (%)	61 (80.3)	74 (46.5)	<0.001
	Albumin, g/dL	2.7 ± 0.56	3.2 ± 0.53	<0.001
	C-reactive protein, mg/dL	13.9 ±8.9	15.4 ± 10.1	0.339
	Lactate dehydrogenase, U/L	912 ± 907	713 ± 1682	0.383
	Creatinine, mg/dL	1.19 ± 0.75	1.3 ± 0.86	0.315
Imaging, n (%)	Ground glass opacities	38 (50)	31 (19.6)	<0.001
	Lobar infiltration	2 (2.6)	58 (36.7)	<0.001
Outcomes, n (%)	Ventilation	46 (63.1)	70 (44.3)	0.015
	Intensive care unit	30 (39.5)	43 (27)	0.054
	Mortality	34 (47.2)	59 (37)	0.146

IQR- Interquartile range

^a^Corticosteroids- >20 mg prednisone per day for 2 weeks or more

Univariate analysis (Table 1), demonstrated that, compared to controls, patients with PJP had a higher incidence of hematologic malignancies (44.7% vs. 28.9%, p = 0.017), and inflammatory diseases (18.4% vs. 6.9%, p = 0.007), and a lower frequency of organ transplantation (9.2% vs. 20.8%, p = 0.028). They were treated more frequently with corticosteroids, (61.8% vs. 22.6%, p < 0.001) chemotherapy (36.8% vs. 19.5%, p = 0.006) and antilymphocyte monoclonal antibodies, mainly rituximab (15.8% vs. 5%, p = 0.008). Fewer were treated with sulfamethoxazole and trimethoprim prophylaxis (6.6% vs. 16.4%, p = 0.038). Patients with PJP had lower body mass index (21 vs. 25, p < 0.001), lower albumin levels (2.7 g/dL vs. 3.2 g/dL, p < 0.001), and greater incidence of lymphopenia (<1000/mcl) (80.3% vs. 46.5%, p < 0.001).

The in-hospital mortality rate (47.2% vs. 37%, respectively, p = 0.15) was similar in both groups. However, the rate of invasive ventilator support was higher among the PJP cohort (61.3% vs. 44.3%, p = 0.015).

In multivariate logistic regression analysis for risk factors (Table 2), PJP was independently associated with Rituximab use (OR 11.47 (95%CI 1.5–87.7), p = 0.019), high-dose steroid treatment (OR 4.39 (95%CI 1.52–12.66), p = 0.006), lymphopenia (OR 8.13 (95%CI 2.48–26.61), p = 0.001), low albumin (OR 0.15 (95%CI 0.04–0.54), p = 0.004) and low BMI (OR 0.80 (95%CI 0.68–0.93), p = 0.018).

**Table 2:** Risk factors for PJP based on multivariate logistic regression analysis^a^.

Risk factor	OR	CI (95%)	P-value
Rituximab	11.47	1.5–87.7	0.019
High dose steroids^b^	4.39	1.52–12.66	0.006
Lymphopenia (<1000/mcl)	8.13	2.48–26.61	0.001
Albumin (per 1-gram increase)	0.15	0.04–0.54	0.004
BMI	0.80	0.68–0.93	0.018

^a^Variables included: age, sex, BMI, medical history, drugs (steroids, chemotherapy, rituximab, antimetabolites), preventive therapy, lymphopenia, albumin);

^b^Equivalent to ≥20 mg of prednisone

Following the identification of rituximab as a major risk factor for PJP, we present the demographic and clinical data of this cohort of patients, including the time frame from last rituximab treatment to PJP onset (Table 3).

**Table 3:** Demographic and clinical data of PJP patients treated with rituximab.

Sex	Age	Diagnosis	Treatment	Prevention	Death	Days since treatment
F	71	DLBCL	CHOP	No	No	90
M	70	DLBCL	CHOP	No	No	36
M	69	DLBCL	CHOP	No	Yes	20
M	42	DLBCL	CHOP	Yes	No	7
M	68	DLBCL	CHOP	No	No	7
M	83	CLL	Steroids	No	No	27
M	60	CLL	Steroids	Yes	Yes	13
F	56	CLL	Bendamustine	No	Yes	50
M	74	AML, ITP	Steroids	No	No	4
M	52	ALL	Cyclophosphamide	No	No	20
F	68	ANCA vasculitis	Steroids	No	Yes	30
F	68	CNS lymphoma	Steroids	No	No	14

DLBCL-Diffuse large B-cell lymphoma; CHOP-Cyclophosphamide, Doxorubicin, Vincristine, Prednisone; CLL-Chronic lymphocytic leukemia; AML-Acute myelocytic leukemia; ITP-Immune thrombocytopenic purpura; ALL-Acute lymphocytic leukemia; ANCA-Antineutrophil cytoplasmic antibodies; CNS-Central nervous system

## Discussion

In this multicenter study of risk factors for PJP in non-HIV patients, antilymphocyte antibodies, mainly rituximab, were found to be the leading risk factor for PJP, with an OR >11. Rituximab, an anti-CD20 antibody was approved in 1997 for B-cell hematological malignancies and autoimmune disorders. In 2007, a black box warning regarding excessive risk for progressive multifocal leukoencephalopathy was issued. Several publications implied a role of rituximab in causing CD4 lymphopenia, although most patients treated with rituximab did not have serial CD4 measurements [17]. Another study on the immunity required for PJP clearance identified a critical role for B cells. In addition to production of PJP-specific antibodies, B-cells are required to prime CD-4 cells for normal expansion and memory generation [18]. The link between rituximab and PJP was reported previously [19]; although, most patients received it as part of combination therapy. Martin-Garrido et al. followed patients treated with rituximab: 30 subsequently developed PJP, but only 3 had been treated with rituximab alone, while the others had received it in combination with either steroids or chemotherapy [19]. In a group of 7,554 rituximab-treated patients in Taiwan, the prevalence of PJP was found to be 2.95%, as compared to 1.3% in controls [20].

In our cohort of 12 PJP patients treated with rituximab, all developed the disease within 90 days of last treatment. This is in accordance with a short report by Alexandre et al. of 11 similar patients who developed PJP within 11 weeks [21]. Although the numbers are small, this might suggest that long term preventive therapy might not be required.

Steroid use (OR 4.98) is a well-known risk-actor for PJP and an indication for preventive therapy [4]. Of note, the odds for developing PJP were twice as high in patients treated with antilymphocyte antibodies than in those treated with steroids. Low BMI and low albumin are proxies for inadequate nutrition, and thus, are expected risk-factors [2].

Mansharamani et al. showed that CD4 <300 denoted a higher risk for PJP infection among immunocompromised patients and recommended they receive prophylaxis [22]. We identified lymphopenia (<1000/mcl) as a risk-factor (OR 7.96), but we did not have CD4 lymphocyte counts for our non-HIV patients.

This study had several limitations. It included patients whose PJP was diagnosed by microscopy only. Although this diagnostic method decreased the number of false positive results and instances of colonization in the case group, it might have increased the incidence of false negative results in the control group. Due to the retrospective nature of the study, data on BMI were incomplete, and HIV status was not ascertained in the HIV-negative individuals. However, information regarding drug therapy, the primary focus of the study, was retrieved from pharmacy records and thus, was complete.

In conclusion, rituximab, which is prescribed for a wide variety of malignant and inflammatory disorders, was found to be significant risk-factor for PJP. Increased awareness of possible PJP infection in this patient population is warranted.

## Supporting information

S1 Table Diseases in cases and controls grouped by mechanism. (DOCX)

S2 Table Drugs used in the case group, grouped by mechanism. (DOCX)
