# Supplementary material for: Rituximab identified as an independent risk factor for severe PJP: A case-control study
Source: PLoS One. 2020 Sep 11;15(9):e0239042. doi: 10.1371/journal.pone.0239042 (PMC7485893; doi:10.1371/journal.pone.0239042)
Supplement: S1 Table — (DOCX) [file pone.0239042.s001.docx]

S1Table. Diseases in cases and controls grouped by mechanism:

| Disease group | Cases(76) | Controls (159) |
| --- | --- | --- |
| Hematologic malignancies | 34 | 46 |
| Lymphoma | 21 | 20 |
| Leukemia | 10 | 23 |
| Multiple myeloma | 3 | 3 |
| Inflammatory diseases | 14 | 11 |
| Anca vasculitis | 1 | 1 |
| Giant cell myocarditis | 1 | 0 |
| Henoch-Schönlein purpura | 1 | 0 |
| Idiopathic pericarditis | 0 | 1 |
| Inflammatory bowel disease | 1 | 1 |
| Interstitial lung disease | 1 | 1 |
| Myofibroblastic tumor | 1 | 0 |
| Optic neuritis | 1 | 0 |
| Poly/dermatomyositis | 2 | 0 |
| Psoriatic arthritis | 0 | 1 |
| Rheumatoid arthritis | 0 | 4 |
| Sarcoidosis | 1 | 0 |
| Scleroderma | 0 | ` 1 |
| Sjogren's syndrome | 1 | 0 |
| Uveitis | 1 | 0 |
| Temporal arteritis | 2 | 1 |
| Solid tumors | 14 | 18 |
| Breast | 1 | 1 |
| Central nerves system | 2 | 1 |
| Larynx | 0 | 1 |
| Lung | 7 | 8 |
| Pancreas | 1 | 0 |
| Prostate | 0 | 1 |
| Renal cell carcinoma | 1 | 1 |
| Salivary gland | 0 | 1 |
| Sinuses | 0 | 1 |
| Stomach | 0 | 1 |
| Thyroid | 0 | 1 |
| Uterus | 1 | 1 |
| Unknown origin | 1 | 0 |
| Solid organ transplant | 7 | 33 |
| Heart | 0 | 1 |
| Kidney | 3 | 7 |
| Lung | 2 | 23 |
| Liver | 1 | 2 |
| Unknown | 1 | 0 |
