# Supplementary material for: Rituximab identified as an independent risk factor for severe PJP: A case-control study
Source: PLoS One. 2020 Sep 11;15(9):e0239042. doi: 10.1371/journal.pone.0239042 (PMC7485893; doi:10.1371/journal.pone.0239042)
Supplement: S2 Table — (DOCX) [file pone.0239042.s002.docx]

S2 Table: Drugs used in the case group, grouped by mechanism

| **Drug group** | Cases (76) | Controls (159) |
| --- | --- | --- |
| Chemotherapy |  |  |
| Bendamustine | 1 | 1 |
| Bleomycin | 1 | 2 |
| Carboplatin | 2 | 2 |
| Carmustine | 1 | 0 |
| Chlorambucil | 0 | 2 |
| Cisplatin | 0 | 5 |
| Cyclophosphamide | 11 | 10 |
| Cytarabine | 2 | 4 |
| Dacarbazine | 1 | 2 |
| Docetaxel | 0 | 2 |
| Doxorubicin | 13 | 13 |
| Etoposide | 1 | 2 |
| Fludarabine | 0 | 1 |
| Gemcitabine | 1 | 1 |
| Hydrea | 0 | 1 |
| Pemetrexed | 1 | 0 |
| Procarbazine | 1 | 0 |
| Temozolomide | 2 | 0 |
| Vinblastine | 1 | 2 |
| Vincristine | 9 | 7 |
| Vinorelbine | 2 | 2 |
| 5FU | 0 | 2 |
| Monoclonal antibodies | 13(16%) |  |
| Alemtuzumab | 1 | 0 |
| Rituximab | 12 | 8 |
| Anti TNF |  |  |
| Etanercept | 0 | 1 |
| Adalimumab | 0 | 1 |
| Kinase inhibitors |  |  |
| Cetuximab | 0 | 1 |
| Crizotinib | 1 | 0 |
| Dasatinib | 0 | 1 |
| Ibrutinib | 0 | 2 |
| Pazopanib | 0 | 1 |
| Sunitinib | 1 | 1 |
| Immunomodulatory/ Proteasome inhibitors |  |  |
| Bortezomib | 0 | 2 |
| Lenalidomide | 1 | 3 |
| Carfilzomib | 1 | 0 |
| Thalidomide | 1 | 0 |
| Immunosuppression |  |  |
| Cyclosporine | 3 | 2 |
| Everolimus | 2 | 1 |
| Mycophenolate mofetil | 4 | 29 |
| Tacrolimus | 5 | 31 |
| Antimetabolite |  |  |
| Methotrexate | 6 | 6 |
| Azathioprine | 2 | 0 |
| Miscellanies |  |  |
| Azacytidine | 0 | 2 |
| Belinatumumab | 0 | 1 |
| Pembrolizumab | 0 | 1 |
| Romidapsin | 1 | 1 |
